# Progress in Pediatrics

**Published:** 1900-11-03

**Authors:** 


### PROGRESS IN PEDIATRICS.

**Laboratory Milk.**—This food supply for infants is employed on a very large scale in America, and it is from America that information as to the results may be expected. Dairies and cows are under careful supervision. The milk is pasteurised and packed in ice. The infant receives from day to day a food uniform in quantity, each feed being in a separate bottle, with an identical percentage of fat, sugar, and proteids as ordered by the physician, and a fixed alkalinity. It is thus, according to Louis Starr,[1] "theoretically the most perfect substitute for normal human milk that science has yet devised, but unfortunately, clinical experience, in my own practice at least, does not bear this theory out." He has seen only three cases in which a state of health was maintained on this diet up to the time of beginning a mixed diet. On the other hand, he has met with sixteen cases where this diet had to be stopped owing to impaired nutrition. The symptoms in these cases were: pallid dry skin, dry lustreless hair, flabby soft muscles, indifferent appetite, sluggish bowels, listlessness and disinclination to play, peevishness, and restless sleep. In thirty-five cases laboratory milk feeding had to be discontinued because of some acute disorder, also of dietetic origin. The explanation of these facts is a matter of great difficulty, and Starr's view is that in the process of preparation the natural emulsion of the milk has been destroyed. For the fat is entirely removed by the separator, and the mixture given is an artificial re-combination of this fat (cream) with an alkaline solution of proteids and sugar. This, according to the writer, may lessen the digestibility of the proteids and lead to a condition either of malnutrition or of irritative diarrhœa. The experience of Starr is very valuable, and may well "give us pause" in this country, where the use of laboratory milk is being pushed by some energetic people.

**The Separation of Bacteria from Milk by Natural Process.**—Rowland Freeman[2] has been investigating the relative proportion of bacteria in milk and cream which have separated by standing. He finds that the cream contains 300 times as many bacteria as the milk, and that with the rising of the cream about 99 per cent. of the bacteria are removed from the milk. This result may be due to the fact that the bacteria grow better in the cream medium than in the milk, and possibly also to the greater supply of oxygen in the former from its position. Or, again, it may be due to the carrying up of the bacteria by the fat globules as they rise, a sort of inverse precipitation. The writer suggests that as some hold that milk heated to 155° Fahr. is impaired as a food material for infants, this difficulty may be partly got over by removing the cream and pasteurising or sterilising it, while the comparatively germ-free milk may be used without being heated.

**Rumination in a Boy.**—The patient was a boy of nine years who, since the age of seven, had been observed to chew and swallow for some time after meals. Luther Peter[3] says that the boy first of all discovered accidentally that he could bring up his food, and, as it tasted the same as before swallowing, he practised the art of rumination so successfully that later the food began to come up without effort. If, however, the regurgitation was induced some time after swallowing, the food had become sour, and he was compelled to spit it out. Careful examination showed that the food had really passed into the stomach and not into any pouch of the œsophagus. The boy grew thin and nervous, and suffered from loss of appetite. By means of careful supervision, the influence of suggestion, and the use of bromides and iron, the habit was broken. There was a history of intemperance on the father's side, and of migraine on the mother's. A personal experience of the condition is recorded by Holliday,[4] who acquired the habit in early childhood and continued it until he learned to smoke at the age of eighteen. He considers that rumination is a neurosis affecting the stomach, not associated with any anatomical change, that males are more frequently affected than females, that it is usually under the control of the will, and that the prognosis as to cure is good.

**Alcoholic Cirrhosis of the Liver in an Infant.**—The following case, related by R. Abrahams,[5] is an example of the occurrence of this affection at a very early period of life. An infant of sixteen months had suffered for eight weeks from intestinal disturbance with enlargement of the abdomen. The abdominal distension was very great, the skin being stretched, glossy, and resistent, and fluid was present in the abdomen. After paracentesis to the extent of a quart of serum, the liver was found to be smooth and hard, and enlarged almost to the crest of the ileum. Six weeks later there had been no recurrence of the ascites, and the liver was diminishing in size. There was no indication of syphilis or tuberculosis or malaria, but it was learned that the infant had been given a glass of lager beer daily, in the belief that it would strengthen her.

**Hypertrophic Dilatation of the Colon in Infants.**—This condition is the subject of a study by Professor Johanessen,[6] who reports several additional cases. The accompanying changes are dilatation of the abdomen, chronic constipation frequently alternating with diarrhœa, and dilatation and hypertrophy of the large intestine, especially in the region of the sigmoid flexure. In severe cases ulceration of the large intestine has sometimes been found post-mortem, but the rectum has generally appeared to be healthy. The hypertrophy of the colon is difficult of explanation in the absence of any definite obstruction. Johannessen points out that in infants the segmoid flexure is very long, and runs transversely from left to right, and then returns from right to left to a point where the rectum begins. This may be due to the fact that in fœtal life the sigmoid flexure forms a reservoir for the meconium and may get greatly distended. If this part of the bowel is greatly enlarged, it changes its place and assumes such a position as to retard the forward movement of the intestinal contents. One distended coil may even press upon and obstruct an adjoining bend of bowel. Another possible factor in bringing about the great dilatation of the bowel met with in these cases is the influence of local nervous or vascular disturbance, as pointed out by Treves. The ulcerations met with in the colon are to be regarded as the result of the retention and decomposition of fæcal matter rather than as a part of the primary disease.

[1] Arch. of Ped., Jan. 1900, and Med. Chron., May, 1900. [2] Scot. Med. Surg. Jour., June, 1900, and Arch. of Ped., Aug. 1899. [3] Pediat., July 15, 1899. [4] New York Med. Rec., 189[illegible]. [5] Pediat., July, 1900. [6] Rev. Mens. des Malad. de l'Enfance, Feb. 1900, and Lancet, May 5, 1900.

